# Partial *PTEN* deletion is linked to poor prognosis in breast cancer

**DOI:** 10.1186/s12885-015-1770-3

**Published:** 2015-12-16

**Authors:** P. Lebok, V. Kopperschmidt, M. Kluth, C. Hube-Magg, C. Özden, Taskin B., K. Hussein, A. Mittenzwei, A. Lebeau, I. Witzel, L. Wölber, S. Mahner, F. Jänicke, S. Geist, P. Paluchowski, C. Wilke, U. Heilenkötter, Ronald Simon, Guido Sauter, L. Terracciano, R. Krech, A. von d. Assen, V. Müller, E. Burandt

**Affiliations:** 1 Institute of Pathology, University Medical Center Hamburg-Eppendorf, Hamburg, Germany; 2 Department of Gynecology, University Medical Center Hamburg-Eppendorf, Hamburg, Germany; 3 Department of Gynecology, Regio Clinic Pinneberg, Pinneberg, Germany; 4 Department of Gynecology, Regio Clinic Elmshorn, Elmshorn, Germany; 5 Department of Gynecology, Clinical Centre Itzehoe, Itzehoe, Germany; 6 Department of Pathology, Basel University Clinics, Basel, Switzerland; 7 Institute of Pathology, Clinical Centre Osnabrück, Osnabrück, Germany; 8 Breast Centre Osnabrück, Osnabrück, Germany

**Keywords:** Breast cancer, *PTEN*, FISH, Prognosis

## Abstract

**Background:**

Deletions of chromosome 10q23, including the *PTEN* (phosphatase and tensin homolog) locus, are known to occur in breast cancer, but systematic analyses of its clinical relevance are lacking.

**Methods:**

We thus analyzed a tissue microarray (TMA) with 2,197 breast cancers by fluorescence in-situ hybridization (FISH) using a *PTEN*-specific probe.

**Results:**

*PTEN* deletions were detected in 19 % of no special type, 9 % of lobular, 4 % of tubular cancers and 46 % in carcinomas with medullary features. 98.7 % of deletions were heterozygous and only 1.3 % were homozygous. *PTEN* deletion was significantly linked to advanced tumor stage (*p* = 0.0054), high-grade (*p* < 0.0001), high tumor cell proliferation (Ki67 Labeling Index; *p* < 0.0001), and shortened overall survival (*p* = 0.0090). *PTEN* deletions were inversely associated with features of luminal type breast cancers (ER/PR positivity; *p* < 0.0001 each, and *CCND1* amplification; *p* = 0.0020). *PTEN* deletions were also strongly linked to amplification of genes involved in the PTEN/AKT pathway such as *MYC* (*p* = 0.0430) and *HER2* (*p* = 0.0065). Remarkably the combined analysis of *MYC*, *HER2*, *CCND1* and *PTEN* aberrations suggested that aberrations of multiple PTEN/AKT pathway genes have a strong additive effect on breast cancer prognosis. While cancers with one of these aberrations behaved only marginally different from cancers with none, disease outcome was markedly worse in cancers with two or more aberrations as compared to those with only one aberration (*p* = 0.0002). In addition, the particularly poor prognosis of patients with *HER2* amplification and *PTEN* deletions challenges the concept of *PTEN* deletions interfering with trastuzumab therapy.

**Conclusion:**

*PTEN* deletion occurs in a relevant fraction of breast cancers, and is linked to aggressive tumor behavior. Reduced *PTEN* function cooperates with *MYC* and *HER2* activation in conferring aggressive phenotype to cancer cells.

## Background

Breast cancer is the most common carcinoma detected in women, accounting for about one fifth of new cancer cases in females [1]. Surgical removal of the cancer represents the standard of care followed by radiation and/or adjuvant therapy in patients considered to be at particular risk for persistent local or systemic disease. Histopathological parameters are of particular importance for assessing tumor aggressiveness. This especially applies for pathological stage (pT), histologic grade, and nodal stage (pN). Although powerful in statistical analyses, these parameters are not sufficient to predict the prognosis of individual patients reliably enough in all cases. Analysis of molecular features in cancer cells bears the potential of providing a better estimation of the prognosis than classical pathological parameters alone [2–4].

The *PTEN* gene at 10q23 encodes a lipid phosphatase that functions as a direct antagonist of phosphatidylinositol 3-kinase and is involved in the regulation of the AKT pathway. Inactivation of *PTEN* leads to constitutively activated levels of AKT, thus promoting cell growth, proliferation, survival and migration through multiple downstream effectors [5]. *PTEN* is one of the most frequently deleted genes in various human cancer types [6], and alterations of *PTEN* were reported to have prognostic relevance in gastric cancer [7], colorectal cancer [8], non-small cell lung cancer [9], diffuse large B-cell lymphoma [10], mesothelioma [11] and prostate cancer [12].

In breast cancer - despite various earlier studies - frequency and relevance of *PTEN* alterations is unclear. The frequency of *PTEN* deletions or reduced expression varies from 4 % to 63 % in the literature [13, 14]. Some studies on 99–151 breast cancer patients have suggested associations between *PTEN* inactivation and poor prognosis [15–17], but this could not be confirmed in other studies involving 212–670 cancers [18–20]. In addition, *PTEN* deletion has been intensively discussed as a potential predictor for failure of anti-HER2 therapy [21–23].

To better understand the clinical relevance of *PTEN* deletions in breast cancer we analyzed more then 2,100 breast cancers with clinical follow-up data. Fluorescence in-situ hybridization (FISH) was applied for *PTEN* analysis because FISH is the gold standard for analyzing DNA copy number changes. Moreover, to better understand the role of *PTEN* deletions in cancers with *HER2* amplification we analyzed a historical cohort of cancers that was collected before anti-HER2 treatments were routinely applied to women with *HER2* positive breast cancer. Our data show that *PTEN* deletion is tightly linked to poor disease outcome and they suggest this also applies to the subgroup of *HER2* positive cancers not treated with trastuzumab.

## Methods

### Breast cancer tissue microarray

A pre-existing tissue microarray (TMA) was used for the purpose of this study [24]. In short, one 0.6 mm core was taken from a representative tissue block from each patient. The tissues were distributed among 6 TMA blocks, each containing 263 to 522 tumor samples. The TMA contained in total 2,197 human breast cancer samples from paraffin-embedded tissue specimens fixed in 4 % neutral buffered formalin. The median patient’s age was 63 (range 26–101) years. The use of the specimens and data for research purposes was approved by local laws (HmbKHG, §12,1) and the local ethics committee (Ethics commission Ärztekammer Hamburg, WF-049/09 and PV3652). According to local laws, informed consent was not required for this study. Patient records/information was anonymized and de-identified prior to analysis. All work has been carried out in compliance with the Helsinki Declaration. Survival data were either obtained from the cancer registry of Basel or collected from the patients attending physicians. Raw survival data were available from 1,982 patients (713 patients with and 1,508 without event (death)). The mean follow-up time was 63 months (range 1–176 months). Tumor size and nodal status were obtained from the primary pathology reports. All slides from the tumors were reviewed by specialized pathologists to define the histologic grade according to Elston and Ellis [25] and the tumor type according to the WHO classification (WHO 2012). Four μm sections of the TMA blocks were transferred to an adhesive coated slide system (Instrumedics Inc., Hackensack, New Jersey) for FISH analysis. Molecular data used in this study were available from previously published studies. These included amplification data obtained by FISH for *HER2*, *CCND1*, and *MYC* as well as expression data obtained by immunohistochemistry for estrogen receptor (ER), progesteron receptor (PR) and Ki67 [24, 26].

### Fluorescence in-situ hybridization

**Fig. 1:**
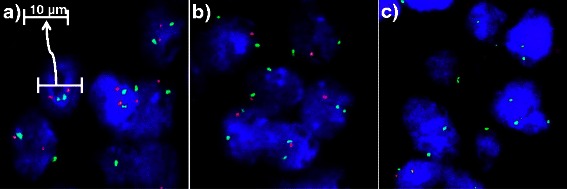
Examples of FISH findings using the *PTEN* deletion probe. **a** Normal *PTEN* copy numbers as indicated by two orange *PTEN* signals and two green centromere 10 signals. b Heterozygous deletion as indicated by the lack of one orange *PTEN* signal and two green centromere 10 signals. **c** Homozygous deletion as indicated by the complete absence of orange *PTEN* signals and the presents of two centromere 10 signals in all tumor cells

Four micrometer TMA sections were used for fluorescence in-situ hybridization (FISH). For proteolytic slide pretreatment, a commercial kit was used (paraffin pretreatment reagent kit; Abbott, Wiesbaden, Germany). TMA sections were deparaffinized, air-dried, and dehydrated in 70 %, 85 %, and 100 % ethanol, followed by denaturation for 5 min at 74 °C in 70 % formamid 2x SSC solution. The home-made FISH probe set consisted of a spectrum-orange labeled *PTEN* probe (made from a mixture of BAC RP11-380G5 and BAC RP11-813O3), and a spectrum-green labeled commercial centromere 10 probe (#6J37-10; Abbott, Wiesbaden, Germany) as a reference. Hybridization was performed overnight at 37 °C in a humidified chamber. Slides were subsequently washed and counterstained with 0.2μmol/L 4′-6-diamidino-2-phenylindole in antifade solution. Stained slides were manually interpreted with an epifluorescence microscope (Axio Imager A1, Zeiss, Oberkochen, Germany), and the predominant FISH signal numbers were recorded in each tissue spot. Presence of fewer *PTEN* signals than centromere 10 probe signals in at least 60 % tumor nuclei were considered as heterozygous deletion. Complete absence of *PTEN* signals in the tumor cells, but presence of centromere 10 signals and *PTEN* signals in adjacent normal cells, was considered homozygous deletion (Fig. 1). Tissue spots lacking any detectable *PTEN* signals in all (tumor and normal cells) or lack of any normal cells as an internal control for successful hybridization of the FISH probe were excluded from analysis. These thresholds were based on our previous study analyzing *PTEN* deletions on a prostate cancer TMA [12].

### Statistics

Statistical calculations were performed with JMP 9 software (SAS Institute Inc., NC, USA). Contingency table analysis and Chi square test were used to study the relationship between IHC and FISH results and clinicopathological variables. Kaplan–Meier plots were used to estimate disease-specific and overall survival and the statistical significance was determined by the log rank test. The log-Rank test was applied to test the significance of differences between stratified survival functions.

## Results

### *PTEN* deletion frequency

A total of 1,239 (56.4 %) of arrayed cancer samples were analyzable by FISH. *PTEN* deletions were found in 233 (19 %) interpretable breast cancers, including 17 % heterozygous and 2 % homozygous deletions.

### Association to breast cancer phenotype

*PTEN* deletions were found in 19 % of NST cancers, 9 % of lobular (*p* = 0.0036 vs NST), 35 % of papillary cancers (*p* = 0.1150 vs NST) and 46 % of cancers with medullary features (*p* = 0.0002 vs NST). If all cancers were jointly analyzed, deletion of *PTEN* were significantly linked to advance tumor stage (*p* = 0.0054), and high histopathological grade (*p* < 0.0001) in all cancers. These associations held also true in the largest subset of No Special Type (NST) cancers. In addition, *PTEN* deletions were strongly linked to the subset of hormone receptor (ER/PR) negative breast cancers: deletion was found in 43 % of ER negative and 24 % of PR negative but only in 11 % of ER negative and 11 % of PR positive breast cancers (*p* < 0.0001 each). *PTEN* deletion was unrelated to presence of lymph node metastases. All results are summarized in Table 1.

**Table 1:** Clinico-pathological association of *PTEN* deletion

			*PTEN* FISH		
		Analyzable (n)	normal	deletion	*P* value
All cancers		1239	81 %	19 %	
Histology	No special type	917	81 %	19 %	-
	Lobular carcinoma	123	91 %	9 %	0.005*
	Cribriform carcinoma	43	88 %	12 %	0.32
	Medullary carcinoma	39	54 %	46 %	0.0001
	Tubular carcinoma	28	96 %	4 %	0.04
	Papillary carcinoma	17	65 %	35 %	0.11
	Mucinous carcinoma	29	97 %	3 %	0.03
	Other rare types^a^	43	58 %	42 %	0.007
pT stage	pT1	411	87 %	13 %	0.005
	pT2	613	78 %	22 %	
	pT3	61	77 %	23 %	
	pT4	146	79 %	21 %	
BRE grade	Grade 1	308	93 %	7 %	<0.0001
	Grade 2	472	87 %	13 %	
	Grade 3	457	68 %	32 %	
Nodal stage	pN0	524	82 %	18 %	0.18
	pN1	446	79 %	21 %	
	pN2	64	72 %	28 %	
ER status	Negative	296	57 %	43 %	<0.0001
	Positive	906	89 %	11 %	
PR status	Negative	744	76 %	24 %	<0.0001
	Positive	408	89 %	11 %	

*Abbreviations*: *pT* Pathological tumor, *BRE* Breast cancer histologic, *ER* Estrogen receptor, *PR* Progesterone receptor, *FISH* Fluorescence in-situ hybridization

^a^: Including adenoid-cystic carcinoma, apocrine carcinoma, atypical medullary carcinoma, carcinosarcoma, clear cell carcinoma, histiocytic carcinoma, lipid rich carcinoma, lipid rich or histiocytic carcinoma, metaplastic carcinoma, neuroendocrine carcinoma, signet ring carcinoma, and small cell carcinoma

*: Versus cancers of no special type

### Association to amplifications of *HER2*, *CCND1* and *MYC*

**Fig. 2:**
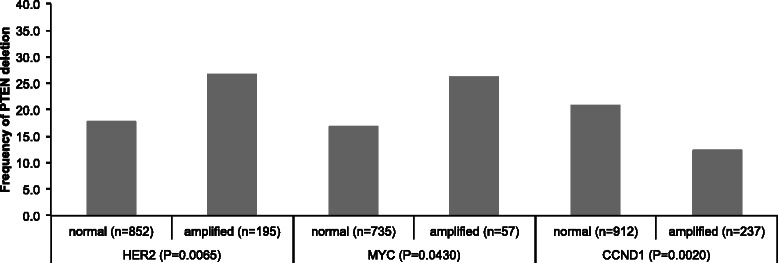
Associations between *PTEN* deletion and amplifications of *HER2*, *MYC* and *CCND1* analyzed by FISH

*HER2*, *CCND1* and *MYC* amplification results were available from a previous study [24]. In total, FISH results on both *PTEN* deletions and amplifications of *HER2*, *CCND1* and *MYC* were available in subsets of 1,047 (*HER2*), 792 (*MYC*) and 1,149 (*CCND1*) cancers. There was a positive association between *PTEN* deletion and amplification of *HER2* and *MYC*, but an inverse association to amplification of *CCND1. PTEN* deletion was found in 27 % of 195 *HER2* amplified cancers but in only 18 % of 852 tumors with normal *HER2* copy numbers (*p* = 0.0065), as well as in 26 % of 57 *MYC* amplified cancers but in only 17 % of 735 tumors with normal *MYC* copy numbers (*p* = 0.0430). In contrast, *PTEN* deletion was found in only 12 % of 237 *CCND1* amplified cancers, but in 21 % of 912 tumors with normal *CCND1* copy numbers (*p* = 0.0020). These associations are shown in detail in Fig. 2.

### Association with cell proliferation

**Fig. 3:**
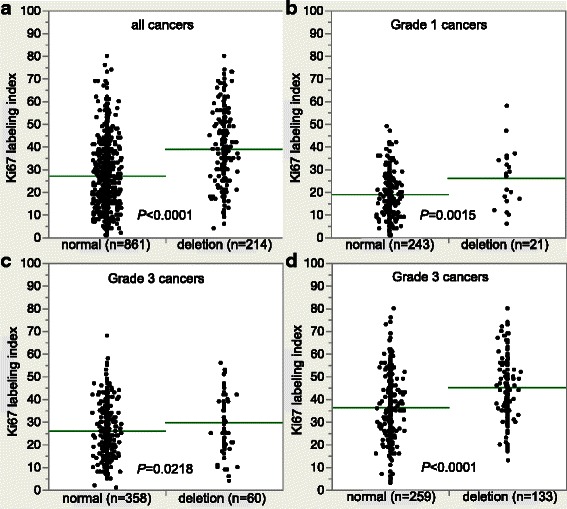
Association between *PTEN* deletion and Ki67-labeling index. **a** all cancers, **b** Grade 1 cancers, **c** Grade 2 cancers and **d** Grade 3 cancers

Data on the tumor cell proliferation, as determined by immunohistochemical analysis of the Ki67 antigen, were available from a previous study using the same TMA [24]. *PTEN* deletions were strongly associated with a high Ki67 labeling index (LI) if all cancers were jointly analyzed (*p* < 0.0001), as well as in subsets of cancers with identical grade (*p* < 0.05 each, Fig. 3a–d).

### Prognostic significance of *PTEN* deletion

**Fig. 4:**
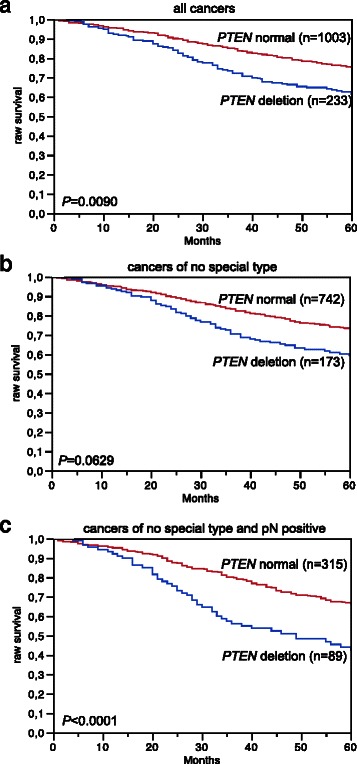
Association between *PTEN* deletion and patient survival. Kaplan Meier plots showing overall (raw) survival in **a** all cancers (*n* = 1,246), **b** no specific type cancers (*n* = 915) and **c** no specific type and pN positive cancers (*n* = 404)

**Fig. 5:**
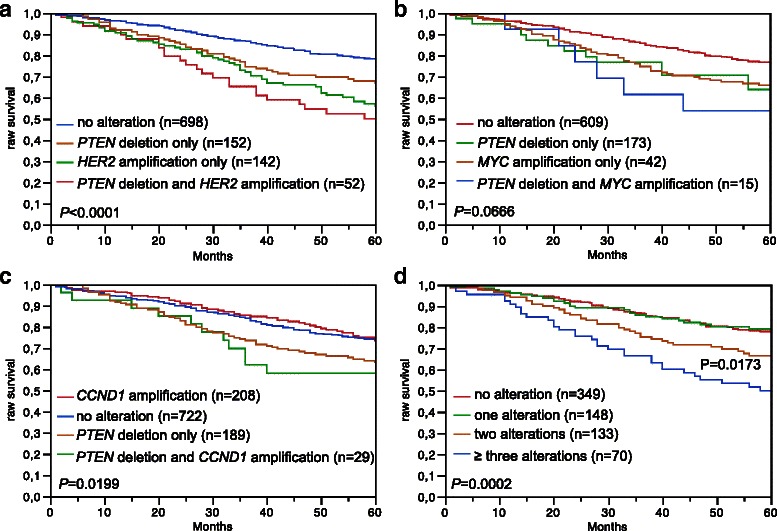
Association between patient survival and co-alterations: Kaplan Meier plots showing overall (raw) survival in cancers with alterations of **a** *PTEN* and *HER2*, **b** *PTEN* and *MYC*, **c** *PTEN* and *CCND1*, and **d** *PTEN*, *HER2*, *MYC* or *CCND1*. No alteration: neither *PTEN* deletion nor oncogene amplification, *PTEN* deletion only: deletion of *PTEN* but no oncogene amplification, oncogene amplification only: oncogene amplification but no *PTEN* deletion, *PTEN* deletion and oncogene amplification: concurrent alteration of both loci

Data on raw survival were available from 1,236 cancers with interpretable *PTEN* FISH results. Presence of *PTEN* deletion was linked to shortened overall survival if all cancers were jointly analyzed (*p* = 0.0090, Fig. 4a), as well as in the subsets of NST cancers (*p* = 0.0629, Fig. 4b), and in the subset of NST cancers with nodal metastases (*p* < 0.0001, Fig. 4c). To study, whether *PTEN* deletion has an additional prognostic value in cancers harboring co-amplification of *HER2*, *MYC*, or *CCND1*, we stratified cancers for survival analysis according to the status of *PTEN* and *HER2* (Fig. 5a), *PTEN* and *MYC* (Fig. 5b), as well as *PTEN* and *CCND1* (Fig. 5c). These analyses suggested an inferior prognosis in cancers harboring *PTEN* deletions with co-amplifications of *HER2*, *MYC*, or *CCND1* as compared to those without co-amplification, but the differences did not reach statistical significance. Since the failure to find significant differences was most likely due to small numbers in the various subsets, we combined all cancers according to the number of alterations, including *PTEN* deletion and amplification of any of *HER2*, *MYC*, and *CCND1*. In this combined analysis, we found a shortened survival for patients with cancers harboring alterations in 2 or more of these genes as compared to tumors with no or one alteration (*p* = 0.0002, Fig. 5d).

## Discussion

Analyzing more than 1,200 informative breast cancers using a FISH probe directed against the known tumor suppressor gene *PTEN* at 10q23, we found that *PTEN* deletion is strongly linked to poor patient prognosis.

*PTEN* deletion was found in 18.8 % of breast cancers. These results are in the range of two earlier studies reporting *PTEN* deletions in 12 %–19 % of unselected breast cancers using FISH [27] or array CGH [16]. In our study, *PTEN* deletion was defined as “fewer *PTEN* signals than centromere 10 signals in at least 60 % of all tumor cells”. These stringent criteria resulted in a 100 % concordance of results found by FISH and comparative genomic hybridization in a previous *PTEN* study of our group in prostate cancer [12]. A higher PTEN deletion rate by FISH (41 %) was only reported from a study on 199 highly selected metastatic breast cancers [28], as well as from heterozygosity (LOH) studies, reporting *PTEN* deletion in 30 %–41 % of 22–105 analyzed breast cancers [29–33]. LOH induced by unequal allele distribution in the case of triploid or aneuploidy cancers or tumors with overrepresentation of chromosome 10 may contribute to the relatively high rates of *PTEN* losses in these studies. FISH represents the gold standard for gene copy number analysis, because FISH is independent of the purity of cancer tissue and chromosomal aberrations such as polysomy. Deletions can be analyzed on a cell-by-cell basis, and abnormalities can be detected in a few cells or even a single cell.

Numerous studies measured alterations of PTEN protein expression by IHC in breast cancer. These studies show highly discordant results, with PTEN loss ranging from 4 %–63 % in 33–670 analyzed breast cancers [13, 14, 18, 31]. Reasons for this variability might include inherent issues of IHC, as no validated antibody, threshold, or protocol is available yet for PTEN expression analysis by immunohistochemistry on FFPE tissue [34]. In a previous study, we tested a series of seven anti-PTEN antibodies, but found all of them unsuitable for reliable measurement of PTEN expression in formalin fixed prostate cancer tissues since no meaningful association was found between the staining level of these antibodies and presence of *PTEN* deletions or tumor phenotype [12]. Discordant results were also reported for RNA expression analysis by RT-PCR. Here studies reported loss of PTEN expression in 20 % of 135 and 77 % of 59 analyzed breast cancers [16, 27]. These discrepancies are not surprising, given that the results of such studies are substantially dependent on the purity of cancer tissue and the PTEN expression levels in normal breast epithelium, as well as in inflammatory or stromal cells.

*PTEN* deletions were unevenly distributed between breast cancer subtypes. Lobular cancers had a particularly low and cancers with medullary features had a markedly high rate of *PTEN* deletions as compared to NST cancers. This is a novel and unexpected observation, given that gene mutations of *PTEN* had been reported at similar rates in lobular (2 %) [35] and NST (2.3 %) carcinomas [36]. The high rate of *PTEN* deletions in cancers with medullary features fits well to the increased proliferation rate, which is one of the hallmarks of this kind of tumor [37]. However, it has been suggested that the favorable clinical course of cancers with medullary features may be strongly driven by a lower tendency for lymphovascular invasion and an activated immune defense that might overrule adverse genetic features [38, 39].

The data of our study show, that – at least in the largest subgroups of NST carcinomas -*PTEN* deletions are strongly linked to features of unfavorable tumor phenotype and to shortened overall survival. While most of the earlier studies on 22–258 breast cancers had also found associations with at least some phenotypic features of aggressive cancer [15–17, 30–33, 40], data were more conflicting with respect to patient outcome. The vast majority of studies analyzed the prognostic impact of *PTEN* alterations in NST cancers by immunohistochemistry [15–20, 41–43]. Half of these studies suggested a prognostic impact of lost or decreased PTEN expression in 99–182 tumors using overall survival, metastasis free-survival, disease-related death or recurrence-free survival as clinical endpoints [15–17, 43]. Other studies could not confirm a significant association between *PTEN* alterations and patient outcome using these clinical endpoints in 97–670 patients [18–20, 41, 42]. Only one study analyzed *PTEN* deletions by FISH in a set of 135 tumors [16]. In line with our analysis, this study revealed a strong link between *PTEN* deletion and reduced metastasis-free interval. Therefore our findings, in combination with the ease of measuring *PTEN* deletions by next generation sequencing methods, strongly prompt for including *PTEN* deletion measurement in future multi-parametrical tests.

*PTEN* deletions were strongly linked to *HER2* and *MYC* gene amplification in our patient set. Both HER2 and MYC are closely connected to AKT/PTEN signaling. MYC is a downstream effector of AKT-depended growth signaling but also controls AKT pathway activity in a negative feedback loop by up-regulation of PTEN [44]. Obviously, impairment of this control loop by deletion of *PTEN* can be expected to provide a growth advantage to cancer cells beyond *MYC* amplification alone. HER2 is one of the receptor tyrosine kinases upstream of PTEN. Since *HER2* amplification strongly activates AKT signaling [45], the additional deletion of *PTEN* might confer a similar growth advantage to *HER2* amplified cells as to *MYC*-amplified cells. In line with this notion, we found at least a strong trend towards an inferior prognosis for *HER2* and/or *MYC*-amplified cancers with co-deletion of *PTEN* as compared to tumors harboring only one of these alterations.

Further in line with an important functional interaction between PTEN and HER2, it has been suggested that *PTEN* inactivation confers resistance to anti-HER2 therapies in cancers harboring *HER2* amplification. Given that studies analyzing the impact of *PTEN* loss on trastuzumab response reported massively conflicting results [46], *PTEN* analysis has not been established as a clinical routine test prior to administration of trastuzumab. Some studies have suggested, that PTEN loss or reduced PTEN expression is sufficient to cause a decreased response to trastuzumab [47–49], whereas other studies found that an additional *PIK3* mutation is required to confer resistance to anti-HER2 treatment [50, 51]. Additional studies did not find a relationship between *PTEN* alterations and sensitivity to trastuzumab [51, 52].

Whether or not *PTEN* alterations are of relevance for response to anti-HER2 therapies is of utmost relevance. Since *PTEN* deletion is found in a subset of 27 % of *HER2* amplified cancers that are eligible for HER2 treatment, it can be estimated that *PTEN* deletion could potentially account for therapy failure in about one quarter of breast cancer patients receiving HER2 inhibitors. It is of note, however, that the generally poor prognosis of patients with the combination of both *PTEN* deletion and *HER2* amplification as determined in a historical breast cancer patient set predating the era of anti-HER2 therapies demonstrates that the poor success of trastuzumab in *PTEN* deleted cancers may also be driven by increased cancer aggressiveness irrespective of therapy response.

The strong association of *PTEN* deletions with *HER2* and *MYC* gene amplifications also fits well with the suggested role of PTEN in maintenance of genomic stability. PTEN is required for double strand breakage (DSB) repair by homologous recombination [53]. Consequently, PTEN has been termed “A new Guardian of the Genome” in reference to the crucial role of the p53 tumor suppressor for genome integrity [54]. Since erroneous DSB repair is one important prerequisite of DNA amplification by fusion bridge breakage [55], it seems likely that *PTEN* deletion contributes to development of gene amplification. Evidence for specific factors (such as for example *PTEN* inactivation) facilitating the development of genomic instability comes also from previous studies by others and us showing a nonrandom accumulation of amplifications of different genomic regions, including *HER2* and *MYC*, in a subset of breast cancers that are considered to show an “amplifier” phenotype [26, 56–59].

It is thus of note, that *CCND1* amplifications were inversely linked to *PTEN* deletions in our study. This finding provides further evidence for the existence of molecularly distinct subsets of breast cancers, one of which may be linked to generalized genetic instability with accumulation of numerous genomic amplifications (including *HER2* and *MYC*) as well as deletions (including *PTEN*), and another one that might develop *CCND1* amplification by a more targeted mechanism. This noting is supported by the fact that *CCND1* amplification is strongly linked to hormone receptor (ER/PR) positive breast cancers [60], while *HER2* and/or *MYC* (like *PTEN* deletions in our study) are more often found in ER-negative than in ER-positive cancers [26, 61]. Such a putative instability-independent amplification mechanism may be driven by the synergistic actions of ER signaling and CCND1 in promoting cell cycle progression [62–64], and may include specific selection of cells with increased *CCND1* copy numbers.

The results of our study demonstrate a marked impact of the deletion of one *PTEN* allele on breast cancer aggressiveness and prognosis. This finding is in line with several in-vivo studies demonstrating that deletion of one *PTEN* allele is sufficient to have substantial impact on cell biology. For example in mouse models, heterozygosity for *PTEN* leads to massively increased susceptibility to multiple tumor types [65–67], increased cell proliferation in the thyroid and prostate gland [65], and has been shown to cooperate with other genetic events, such as *ERG* fusion in murine prostate cancer [68, 69]. In an earlier study of 2,266 prostate cancers we had been able to demonstrate that no clinical difference existed between 225 patients with biallelic and 152 patients with monoallelic deletion of *PTEN* [12]. It was traditionally thought that chromosomal deletion is a mechanism to inactivate tumor suppressor genes in a two hit model for example in combination with a mutation of the other allele. However, large scale next generation sequencing of breast cancer genomes is increasingly demonstrating that mutations of “classical” tumor suppressor genes do often not accompany large deletions, suggesting that large deletions may not only serve for inactivation of a single gene [70]. Increasing evidence suggests that deletions impact affected cells by reduced dosage of multiple different genes residing on a certain chromosomal area. Studies in prostate cancer have demonstrated that various chromosomal deletions have marked prognostic relevance [12, 71–74]. As deletions are particularly frequent in breast cancer, it will be interesting to evaluate their clinical potential. This is all the more true, as deletions can relatively easy be studied by NGS even from formalin fixed material [75].

## Conclusions

Our data identify *PTEN* deletions as a frequent event in breast cancer with marked prognostic impact. Given the strong association with *HER2* amplification, and the putative role of *PTEN* inactivation for HER2 therapy failure, our findings prompt for further analysis to estimate the predictive value of *PTEN* deletion analysis for HER2 treatment success in breast cancer.

## Abbreviations

BRE: Breast cancer histologic grade
ER: Estrogen receptor
FISH: Fluorescence in-situ hybridization
PR: Progesterone receptor
pT: Pathological tumor stage
PTEN: Phosphatase and tensin homolog
pN: Nodal stage
TMA: Tissue microarray
